# Identification of miR-4510 as a metastasis suppressor of gastric cancer through regulation of tumor microenvironment via targeting GPC3

**DOI:** 10.1007/s10585-021-10143-6

**Published:** 2022-01-20

**Authors:** Hai-fen Ma, Peng Shu, Xiao-hai Shi, Min Wang, Mao-fen Jiang

**Affiliations:** 1 Department of Pathology, Beilun People’s Hospital, 1288 Lushan East Road, Ningbo, 315800 Zhejiang Province China; 2 Molecular Laboratory, Beilun People’s Hospital, Ningbo, 315800 Zhejiang Province China; 3 Department of Gastrointestinal Surgery, Beilun People’s Hospital, Ningbo, 315800 Zhejiang Province China

**Keywords:** miR-4510, Glypican-3, Gastric cancer, Glypican3-expressing gastric cancer, Tumor microenvironment, Oxaliplatin

## Abstract

The genes miR-4510 and glypican-3 (GPC3) have reported to be closely associated with tumors, with miR-4510 inversely correlated with GPC3 mRNA and protein in hepatocellular carcinoma samples. Glypican-3-expressing gastric cancer (GPC3-GC), characterized as gastric cancer (GC) expressing GPC3, accounts for 11% of the GC cases. However, the expression and mechanism of action of miR-4510 in GPC3-GC have not been clearly defined. We found that miR-4510 expression in GC tissues was significantly lower than that in the adjacent tissues (p < 0.001). miRNA-4510 expression in GPC3-GC was significantly lower than that in GPC3‐negative GC tissue (p < 0.001). Our study confirmed that miR-4510 is inversely correlated with GPC3 in gastric cancer samples and that GPC3 is a direct target gene of miR-4510. The proportion of M2 macrophages in GC with low expression of miR-4510 was significantly increased, while the proliferation of CD8+ T cells was limited. miR-4510 may change the immunosuppressive signals in the tumor microenvironment by downregulating GPC3 and inhibiting gastric cancer cell metastasis. Oxaliplatin treatment may become a specific therapeutic drug for patients with miR-4510 inhibition and GPC3-GC.

## Introduction

Gastric cancer is the fourth most common malignant tumor worldwide [1]. There are about 1 million newly diagnosed gastric cancer patients in the world every year, 47% of them being from China. Most of them already have metastasis at the time of diagnosis [2]. The 5 year survival rate of these patients is only 31%, which is significantly lower than that of patients without metastasis (5 year survival rate is 68%) [3]. Thus, the identification of driver genes in the metastasis process of gastric cancer is urgently needed.

Glypican-3 (GPC3), also known as DGSX, GTR2-2, or MXR7, belongs to the heparin sulfate family, which is anchored on the surface of the cell membrane through phosphatidylinositol [4]. GPC3 plays a critical role in cell proliferation and malignant transformation [5]. The function of GPC3 is mainly linked to the Wnt (Wingless-related integration site ligands), Hedgehog, bone morphogenetic proteins, and fibroblast growth factor signaling pathways [6]. In a study of 926 cases of gastric cancer, glypican3-expressing gastric cancer (GPC3-GC) was characterized as gastric carcinoma (GC) expressing GPC3 with their related entities: hepatoid, clear‐cell, and α‐fetoprotein‐producing GC, and defined as focal GPC3‐GC when 10–49% of neoplastic cells were positive, and as diffuse GPC3‐GC when more than 50% of the cells were positive [7]. Among 926 GC cases, 101 (11%) were GPC3‐GC and both diffuse and focal GPC3‐GC showed nodal metastasis more frequently (67% and 55%, respectively) than GPC3‐negative GC (34%), suggesting that GPC3 acts as an oncogene in gastric cancer [7]. In addition, miR-4510 had been proved as a tumor suppressor in various of cancers, in gastrointestinal stromal tumor, expression of miR-4510 was lower in the tumor tissue compared with the paired adjacent tissue [8]. It was discovered that miR-4510 inversely correlated with GPC3 mRNA and protein levels in hepatocellular carcinoma (HCC) samples. This miRNA also induces apoptosis of hepatoma cells and blocks tumor growth in vivo. The study further showed that the tumor-suppressive effect of miR-4510 was mediated through direct targeting of GPC3 mRNA and inactivation of Wnt/β-catenin transcriptional activity and signaling pathways [9]. However, the expression and mechanism of action of miR-4510 in GPC3-GC have not been clearly defined. Therefore, in this study, we focused on the role of miR-4510 in the occurrence and development of GPC3-GC.

## Material and methods

### Patients’ samples

All gastric cancer tissues were collected from Ningbo Beilun People’s Hospital (Zhejiang, China), and all sample extraction protocols followed the Declaration of Helsinki. Ethical approval was obtained from the hospital, and informed consent was obtained from all the patients before the study. The histological type was primary gastric adenocarcinoma. The diagnosis of GPC3-GC was based on the criteria used by Ushiku et al. [7]. All the tissues were obtained after surgery and fixed in 10% neutral formalin and embedded in paraffin after dehydration. All the study results were blinded to the review by 2 pathologists.

### Cell line

The gastric cancer cell lines used in this experiment, including RAW264.7, AGS, SNU-5, SNU-1, NCI-N87, KATO III, SNU-16, and MFC, were purchased from the Chinese National Infrastructure of Cell Line Resource (NICR); the culture medium was DMEM + 10% FBS, and culture conditions were 37 °C and 5% CO_2_.

### Laboratory mice

The nude mice and 615 mice in this study were purchased from Beijing Vital River Laboratory Animal Technology Co., Ltd. The nude mice are characterized by thymic deletion (low T cell function). The 615 mice have more immune deficiencies than the nude mice, widely used for anticancer drugs creening and immunotherapy and mechanism research of tumors. The animal protocol was designed to minimize pain or discomfort to the animals. The mice were acclimatized to laboratory conditions (23 °C, 12 h/12 h light/dark, 50% humidity, ad libitum access to food and water) for 2 week prior to experimentation. All mice were euthanized by barbiturate overdose (intravenous injection, 150 mg/kg pentobarbital sodium) for tissue collection. The mice were divided into seven groups, 5 for each group. (1) Three groups of the nude mice were injected with SNU-5 Scramble, miR-4510 inhibitor and miR-4510 inhibitor + GPC3-shRNA cells through tail vein injection respectively, each mouse with 1 million. After 4 weeks of injection, the mice were dissected, and lung metastasis was detected using a fluorescence microscope. (2) To detect the change of immune cells in the primary tumor, three groups of 615 mice were injected subcutaneously MFC Scramble, miR-4510 inhibitor and miR-4510 inhibitor + GPC3 overexpressing (OE) cells respectively, each mouse with 5 million, 2 weeks later, collected the primary tumors, digested in single cells, and carried out further experiments. (3) Another five 615 mice were injected subcutaneously with 5 million MFC miR-4510 inhibitor + GPC3 OE cells, one week later, treated with oxaliplatin intraperitoneally at the dose of 20 mg/kg, twice a week, for 2 weeks, then the primary tumors were collected.

### QPCR (quantitative reverse transcription PCR)

(1) mRNA extraction: the tissue was ground and dissolved directly in Tri-zol reagent, centrifuged to remove cells and debris, and mixed with chloroform. The RNA solution was obtained by drawing the aqueous phase. Finally, the solution was mixed with isopropanol to precipitate the RNA, which was washed twice with alcohol and quantified by A260 nm/A280 nm. (2) Total RNA was reverse transcribed using the RevertAid H Minus First Strand cDNA Synthesis Kit (K1632). LightCycler® 480 Probes Master (04887301001) was used to detect the encoding genes using FastStart Universal SYBR Green Master (4913914001, Sigma-Aldrich) to detect the expression of genes at the mRNA level. Primer sequences used in qPCR included GAPDH: F-5′- CATCACTGCCACCCAGAAGACTG-3′, R-5′- ATGCCAGTGAGCTTCCCGTTCAG-3′. ARG1: F-5′- CATTGGCTTGCGAGACGTAGAC-3′, R-5′- GCTGAAGGTCTCTTCCATCACC-3′, MRC-1(CD206): F-5′- GTTCACCTGGAGTGATGGTTCTC-3′, R-5′- AGGACATGCCAGGGTCACCTTT-3′, RETNLA(FIZZ1): F-5′- CAAGGAACTTCTTGCCAATCCAG-3′, R-5′- CCAAGATCCACAGGCAAAGCCA-3′. For miRNA detection, U6 was used as an internal control, and for the detection of gene expression, GAPDH was used as an internal control. The gene expression was quantified by the 2^−△△CT^ method.

### Cell apoptosis, proliferation and migration experiment

The cultured cells were first digested as single cells. Then the following steps were carried out: (1) cell apoptosis: After being washed twice with PBS, cells were suspended in Annexin V binding buffer, and labeled with Annexin V FITC antibody for 15 min at room temperature, after which the cells were labeled with PI and the change in apoptosis was detected using a FACS calibur machine. (2) Cell proliferation: single cells were disseminated in a 96-well plate, 1000 cells were assigned to each well, and 1/10 volume of CCK8 solution was added. Then, cell proliferation at time points 0, 24, 48, 72, and 96 h was detected using a microplate reader. Finally, the proliferation index was represented by OD 450 nm at other time points and OD 450 nm at the 0 h time point. (3) Cell migration: cells were seeded in a 24-well plate, and each well contained 100,000 cells. After cell attachment, we used white tips to scratch the bottom and took pictures of the at 0 h and 12 h.

### Western blot

(1) The tissue was ground and dissolved in radioimmunoprecipitation assay buffer (RIPA) buffer, mixed with loading buffer, and boiled for 10 min. (2) The solution was loaded on an SDS-PAGE gel, and electrophoresis was performed at a constant voltage until bromophenol blue reached the bottom. (3) The separated proteins were transferred to a polyvinylidene fluoride (PVDF) membrane through electrorotation and blocked with 5% bovine serum albumin for 2 h at room temperature. (4) The PVDF membrane was labeled with the primary antibody overnight at 4 °C and labeled with horseradish peroxidase (HRP)-conjugated secondary antibody for 30 min at room temperature. The antibodies used in this experiment included glypican 3 antibodies (Thermo Fisher, MA5-17083) and GAPDH (ab9485, Abcam, Cambridge, UK). (5) The PVDF membrane was then treated with an enhanced chemiluminescence (ECL) kit to quantify the protein expression.

### Luciferase experiment

In the luciferase assay, the cells were transfected with the miR-4510 mimic vector. At the same time, cells were transfected with control vector (pmirGLO Dual-Luciferase miRNA Target Expression Vector, Cat # E1330, Promega Company), original GPC3 3-UTR, and mutation type of GPC3 3-UTR. After 48 h of transfection, cells were lysed and luciferase intensity was detected using a LightSwitch Dual-Luciferase assay kit (Biotek, Winooski, VT, USA) using a microplate reader. Finally, luciferase intensity was normalized by Cypridina TK control, and the fold change of the empty vector was used to express the relative intensity of luciferase.

### CyTOF (cytometry by time-of-flight) experiment

(1) The expression of LY6G, CD8A, CD45, CD11B, CD28, CD3E, LY6C, CD19, CD24, CD14, F4/80, and CD326 in the single cells that were digested from the primary tumor of MFC tumor-bearing mice were detected using CyTOF. (2) Raw data of the CyTOF machine was saved as an FCS file and read in R 4.0.2 language using the flowCore package, and the Rtsne package was used to identify the immune cell population in the primary tumor.

### IHC (immunohistochemistry)

(1) The specimens of 32 cases of gastric cancer tissues (divided into miR-4510 low group and miR-4510 high group) were sectioned into 4-μm thick slices, deparaffinized in 2-xylene, passed through 3 changes of 95% ethanol, and transferred to water. (2) After antigen retrieval, endogenous peroxidase activity was blocked by incubation with 0.3% H_2_O_2_, followed by washing thrice with PBS. (3) The slides were incubated with the primary antibodies F4/80, GPC3, and N-cadherin, followed by incubation with the secondary antibody and chromogen. (4) The expression of the markers was identified at the protein level.

### FACS (fluorescence-activated cell sorting) analysis and T cell proliferation

(1) First, the 615 mice were dissected, and single cells were extracted from the spleen using grinding and erythrolysis. (2) CD8+ T cells were sorted from single cells using a BD FACSAria™ III Cell Sort machine. (3) T cells were placed in a 96-well plate, and each well had 100,000 cells. Then, we added CD3/CD28 dynabeads T cells activation and expansion beads (ThermoFisher, 11132D), and 100,000T cells were saved without antibody group, and all the T cells were labeled with carboxyfluorescein succinimidyl ester (CFSE). (4) Macrophage cells (CD45 + CD11B + CD68 +) were obtained from the single cells above which were digested from the primary tumor of MFC tumor-bearing mice using a FACS sorting machine. The antibodies used in this study were purchased from BioLegend. (5) T cells were co-cultured with macrophages, after 4 days, using a FACSCalibur machine to detect changes in CFSE. (6) To identify the changes in M2 macrophages in primary tumors, we labeled the single cells obtained from the primary tumors with CD45, CD11B, and F4/80 antibodies, and used a FACS Aria III machine to define the percentage of F4/80 positive cells in CD45 + CD11B + cells.

### Drug treatment

For treatment, 14 anticancer drugs were selected. The IC50 of these drugs was confirmed on the cancerrxgene website (www.cancerrxgene.org_data). Then, different concentrations of drugs around the IC50 were used to treat the MFC cells for 48 h. The IC50 of drugs was calculated using the IC50 package in R. Oxaliplatin, purchased from Selleckchem (S1224), was determined to be the most effective chemical component to inhibit miR-4510.

### Statistical analysis

Statistic alanalyses were performed using the SPSS 21.0 software. The significance levels in this study were determined using the Student’s t-test: *p < 0.05, **p < 0.01, ***p < 0.001.

## Results

### Expression of miR-4510 in gastric cancer and the relationship with tumor biological function

**Fig. 1:**
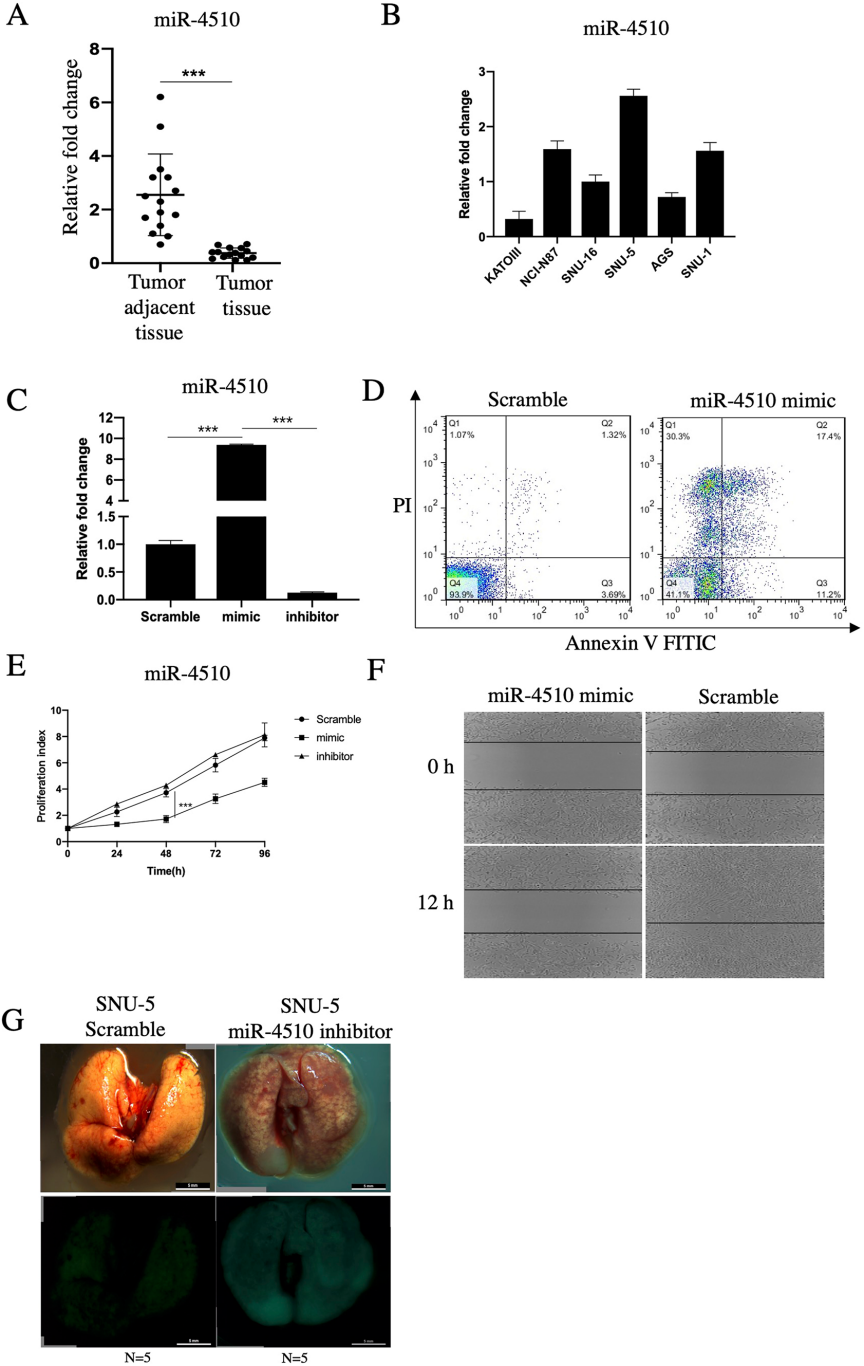
Expression of miR-4510 in gastric cancer and the relationship with tumor biological function. **A**, **B** QPCR was used to detect (**A**), the expression of miR-4510 in 15 cases of gastric adenocarcinoma and tumor adjacent tissue, (**B**), the expression of miR-4510 in 6 types of gastric cancer cell lines. **C**, **D** Scramble, miR-4510 mimic, miR-4510 inhibitor were transfected into KATO III cell line, then **C** expression of miR-4510 in 3 cell lines checked by QPCR, **D** using Annexin V/PI double staining to check for changes in cell apoptosis. **E** Detection of cell proliferation by using CCK8 assay. **F** Using wound healing assay to detect the change of cell migration. **G** In vivo metastasis by injection of SNU-5 Scramble, miR-4510 inhibitor cell lines into the tail vein of nude mice

Based on our previous investigation, the expression of miR-4510 was correlated with GPC3 in liver cancer. Given that GPC3 is a well-known oncogene in hepatocellular carcinoma [9], we concluded that miR-4510 might act as a tumor suppressor in Glypican3-expressing gastric cancer. In this study, we first chose 15 cases of paired gastric cancer tissue and adjacent tissue using QPCR and found that the expression of miR-4510 in the tumor and adjacent tissues was − 2.55 ± 1.52 vs 0.38 ± 0.19 (p < 0.001), and miR-4510 in gastric cancer tissue was significantly lower than in adjacent tissue (Fig. 1A). In the meantime, we thoroughly screened 6 types of stomach cancer cell lines, and the results showed that expression of miR-4510 was highest in SNU-5 and lowest in KATOIII cell line (Fig. 1B). The miR-4510 mimic and miR-4510 inhibitor were transfected into KATO III cell lines (Fig. 1C), then we found that the overexpression of miR-4510 in KATO III cell lines could induce cell apoptosis (Fig. 1D), inhibit cell growth (Fig. 1E), and further downregulate the migration ability of gastric cell lines (Fig. 1F). Tumor metastasis accounts for most cancer-related deaths in the development of gastric cancer. Through tail vein injection of SNU-5 miR-4510 inhibitor cells in nude mice, we further confirmed that miR-4510 inhibition could significantly increase the metastatic ability in vivo (Fig. 1G).

### GPC3 served as a direct target of miR-4510

**Fig. 2:**
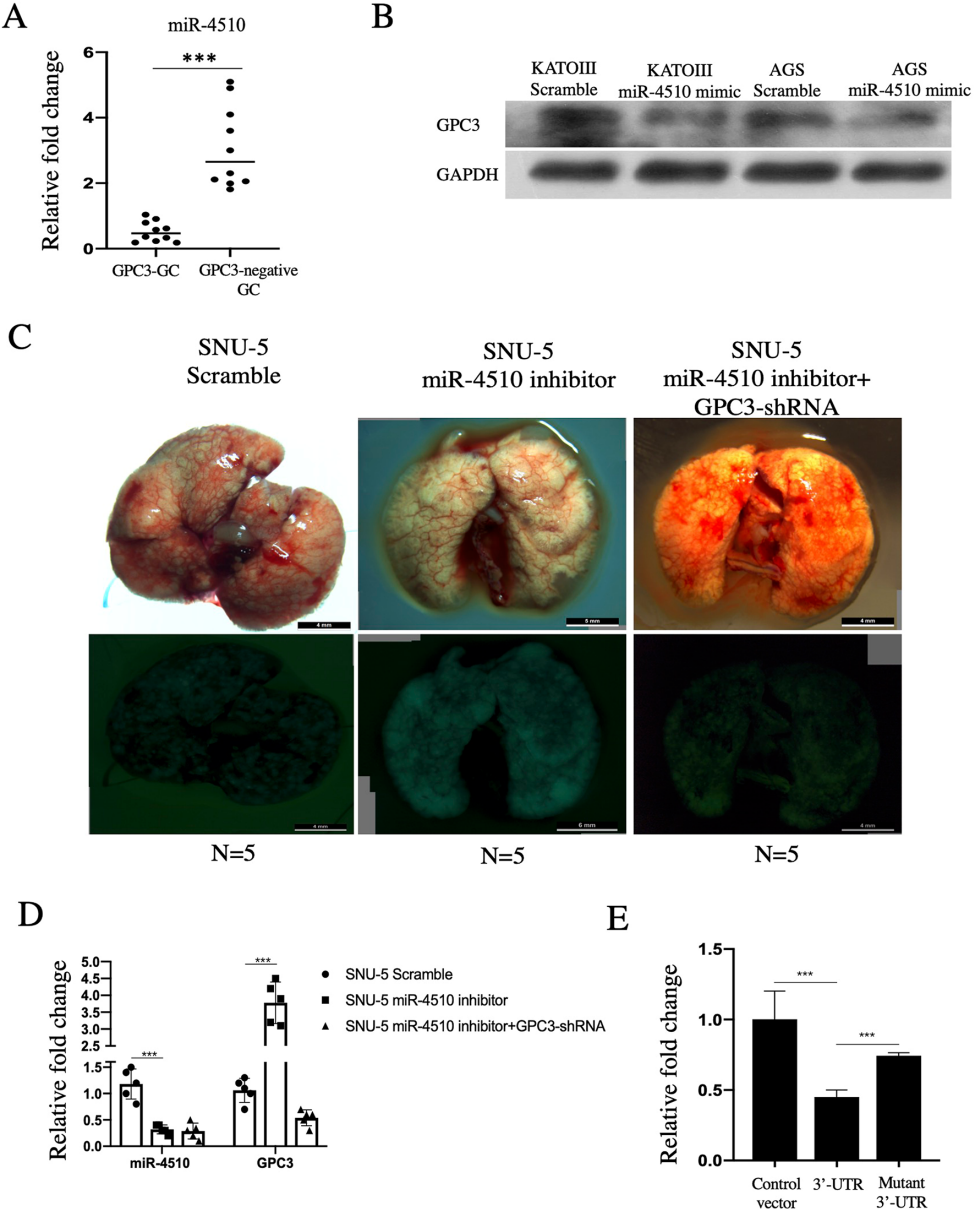
GPC3 served as a direct target of miR-4510. **A** In 10 cases of GPC3-GC tissue and 10 cases of GPC3-negative GC tissue, QPCR was used to detect the change in miR-4510. **B** KATO III and AGS cell lines were transfected with Scramble and miR-4510 mimic for 48 h, and the expression of GPC3 was detected at the protein level. **C** SNU-5 cells were transfected with Scramble, miR-4510 inhibitor and miR-4510 inhibitor + GPC3 OE for 48 h, and then injected in the tail vein of nude mice. **D** Green fluorescent protein (GFP) + cells were sorted from the lung of Scramble, miR-4510 inhibitor and miR-4510 inhibitor + GPC3 OE mice, and the expression of miR-4510 and GPC3 was detected. **E** SNU-5 cells were transfected with CON, normal GPC3-3′-UTR, mutant GPC3-3′-UTR, and the change in luciferase intensity was detected

We then attempted to define the relationship between GPC3 and miR-4510 expression. In 10 cases of GPC3-GC and 10 cases of GPC3‐negative GC tissue, expression of miR-4510 was 0.52 ± 0.31 vs 3.10 ± 1.25 (p < 0.001), which indicates the correlation between GPC3 and miR-4510 (Fig. 2A). We also selected KATO III and AGS cell lines because of the high intrinsic expression of GPC3, transfected with the miR-4510 mimic, and the results showed that GPC3 was downregulated by miR-4510 overexpression (Fig. 2B). Furthermore, by tail vein injection, the elevated tumor metastasis could be softened by miR-4510 inhibition + GPC3-shRNA, suggesting that the function of miR-4510 relied on GPC3 (Fig. 2C, D). Through luciferase experiments, we confirmed that GPC3 acted as a target of miR-4510 (Fig. 2E).

### *Increase of M2 macrophage induced by miR-4510 inhibition *in vivo

**Fig. 3:**
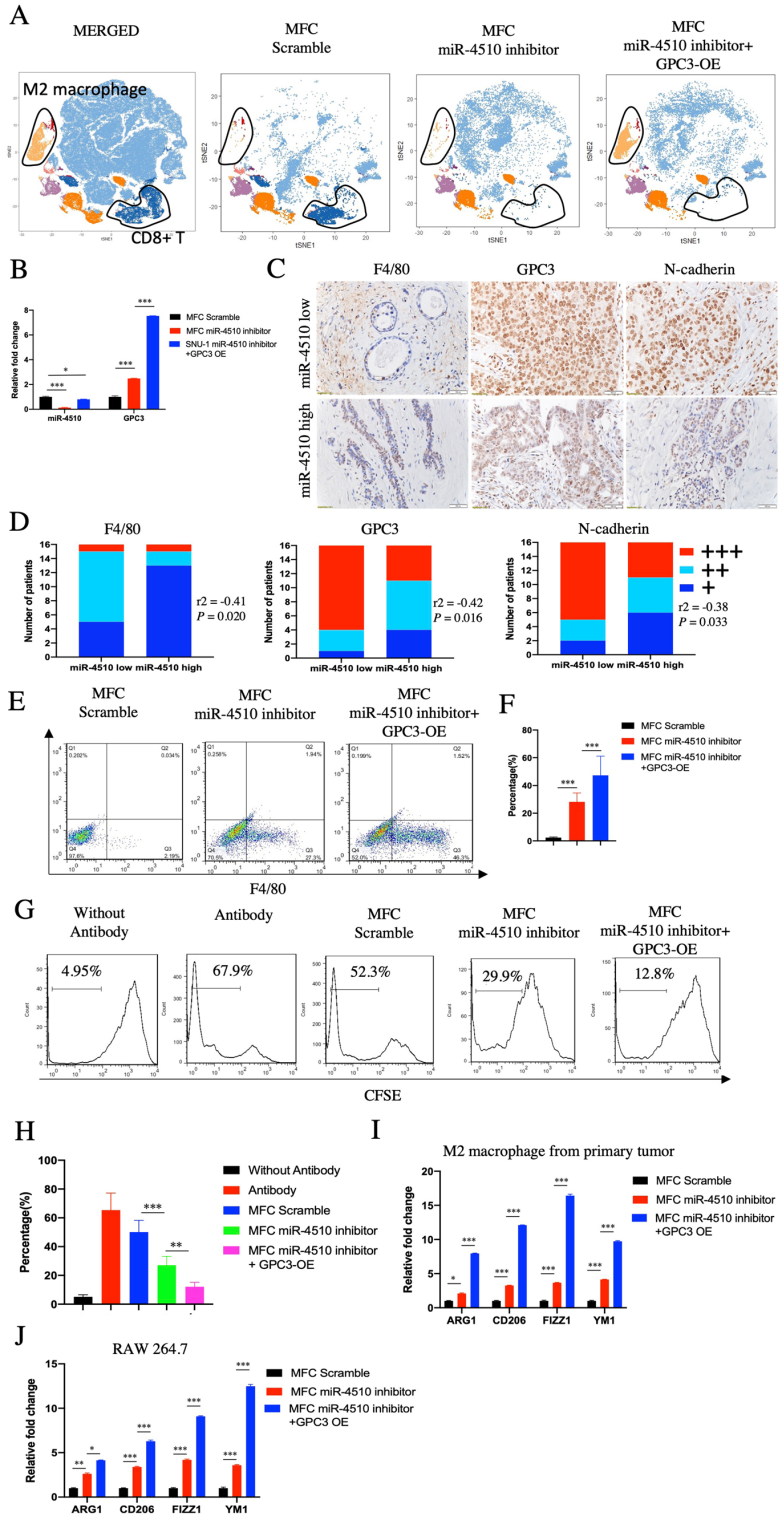
Increase in M2 macrophage induced by miR-4510 inhibition in vivo. **A** MFC Scramble, miR-4510 inhibitor and miR-4510 inhibitor + GPC3 OE were injected in 615 mice subcutaneously, followed by CyTOF to detect the profile of immune cells in primary tumor. **B** Using QPCR to detect the expression of miR-4510 and GPC3 in 615 mice MFC Scramble, miR-4510 inhibitor and miR-4510 inhibitor + GPC3 OE cells. **C** Expression of F4/80, GPC3 and N-cadherin in 32 cases of gastric cancer tissues (divided into miR-4510 low group and miR-4510 high group) were identified by IHC. **D** Quantification of (**C**). **E** Change in M2 macrophage in CD45 + CD11B + cells from primary tumor. **F** Representative summary from (**E**). **G** Firstly, CD8+ T cells of spleen were sorted, and divided into (1) without antibody (CD8 + T cells without treatment by CD3, CD28 antibody and cultured alone), (2) antibody (CD8+ T cells treated with CD3, CD28 antibody), (3) Scramble (CD8+ T cells treated with CD3, CD28 antibody and co-cultured with macrophage from MFC scramble primary tumor), (4) miR-4510 inhibitor (co-culture with miR-4510 inhibitor primary tumor), (5) miR-4510 inhibitor + GPC3 OE (co-culture with miR-4510 inhibitor + GPC3 OE primary tumor). **H** Representative summary from (**G**). **I** macrophage from MFC Scramble, miR-4510 inhibitor, miR-4510 inhibitor + GPC3 OE primary tumor was sorted, and expression of ARG1, CD206, FIZZ1, YM1 were detected. **J** RAW 264.7 were treated with condition medium from MFC Scramble, miR-4510 inhibitor, miR-4510 inhibitor + GPC3 OE for 24 h, and expression of ARG1, CD206, FIZZ1, YM1 were identified

GPC3 is a well-known oncogene that regulates cell proliferation and differentiation. In gastric cancer, GPC3 is not only highly expressed in the tumor tissue but is also related to its clinical stage, Lauren classification, and prognosis [7]. The mechanism of GPC3 is related to the activation of BMP7, Wnts, and insulin-like growth factor (IGF) signaling. Recent studies have shown that the function of GPC3 can also be linked to the tumor microenvironment. In GPC3-GC tissue, polarization of macrophages from M1 to M2 was induced, which resulted in an increase in M2 macrophages [10]. In our study, we chose 615 mice because of the complete microenvironment of these mice [11]. First we injected them with the MFC scramble, miR-4510 inhibitor and miR-4510 inhibitor + GPC3 OE cells, followed by CyTOF. We confirmed that in the primary tumors developed in the MFC miR-4510 inhibitor mice, M2 macrophages were slightly increased, but the proportion of CD8+ T cells was significantly decreased. After GPC3 was overexpressed, M2 macrophages were further increased, and CD8+ T cells were also lower than those in the scramble group (Fig. 3A). At the same time, using qPCR to detect the expression of miR-4510 and GPC3, we found that GPC3 was slightly increased, but miR-4510 was significantly decreased in the primary tumors from the MFC miR-4510 inhibitor mice. GPC3 further increased, and miR-4510 expression was lower than that in the scramble group (Fig. 3B). These results were repeated using IHC and FACS, which showed lowered expression of miR-4510 in the primary tumors. M2 macrophages were increased (Fig. 3C–F), and the invasive marker N-cadherin was increased in the miR-4510 low expression group, suggesting that the immune suppressive signal was activated in tumor tissues with miR-4510 low expression. In the meantime, we performed T cell proliferation experiments and found that macrophages from the miR-4510 inhibitor primary tumor successfully inhibited CD8+ T cell proliferation in vitro, and overexpression of GPC3 further induced this effect (Fig. 3G, H). Expression of activated markers of M2 macrophages was also elevated in the M2 macrophages sorted from the miR-4510 inhibitor primary tumor, or RAW 264.7 cell line treated with conditioned medium from miR-4510 inhibitor cells (Fig. 3I, J). Combining these results, miR-4510 inhibition could elevate the percentage and function of M2 macrophages.

### Reversing of miR-4510 by oxaliplatin treatment

**Fig. 4:**
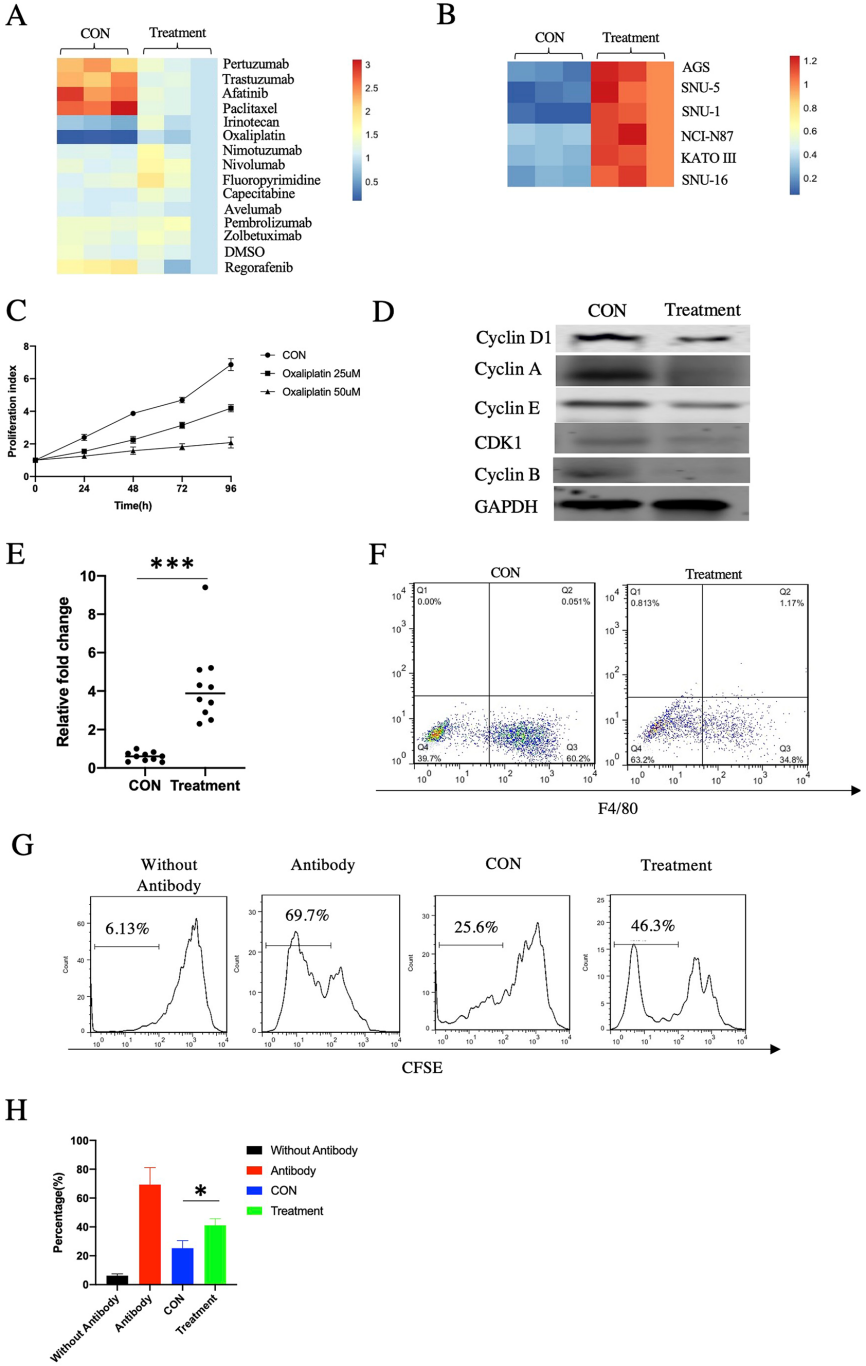
Reversing of miR-4510 and GPC3 by oxaliplatin treatment. **A** Expression of miR-4510 in MFC cells which were treated or untreated with various drugs for 48 h. **B** Expression of miR-4510 in AGS, SNU-5, SNU-1, NCI-N87, KATO III, SNU-16 cells which were treated or untreated with 25 uM oxaliplatin for 48 h. **C** Cell proliferation of CON, oxaliplatin 25 uM and oxaliplatin 50 uM group cells. **D** Using western blot to detect the change of cell proliferation markers after treatment with oxaliplatin. The 615 mice were injected with MFC miR-4510 inhibitor + GPC3 OE cells, and treated or untreated with oxaliplatin, followed by (**E**) QPCR to detect the expression of miR-4510 in primary tumor, **F** FACS to identify the percentage of F4/80 in CD45 + CD11B + cells from primary tumor. **G** Proliferation of CD8+ T cells was detected after co-culturing with macrophage from before and after treatment of mice primary tumor. **H** Representative summary of (**G**)

We then tried to inhibit the metastasis correlated with miR-4510 through drug treatment. We firstly screened 14 drugs commonly used in clinic, the statistical results indicated that there was asignificant difference only in oxaliplatin type (p < 0.001) (Fig. 4A). oxaliplatin could increase the expression of miR-4510 in MFC cells, suggesting that oxaliplatin could reverse the expression of miR-4510. Treatment with oxaliplatin also changed the expression of miR-4510 in AGS, SNU-5, SNU-1, NCI-N87, KATO III, and SNU-16 cells (Fig. 4B). In addition, oxaliplatin inhibited the proliferation of MFC cells (Fig. 4C), as well as the expression of cell growth markers (Fig. 4D). Furthermore, we collected the primary tumors from the 615 mice that were injected with MFC miR-4510 inhibitor + GPC3 OE cells and then treated with oxaliplatin. We confirmed that in the oxaliplatin-treated mice, expression of miR-4510 (Fig. 4E) was elevated and that of the M2 macrophages (Fig. 4F) was decreased in the treatment group using QPCR. CD8+ T cell proliferation was also suppressed after co-culturing with macrophages in the treatment group (Fig. 4G). Combining these results, we proved that oxaliplatin treatment could reverse the expression of miR-4510, which further suppressed the function of M2 macrophages.

## Discussion

miRNAs are a class of endogenous non-coding small RNA molecules with a length of approximately 17–25 nucleotides. Mutations and abnormal expressions in miRNAs are related to the occurrence and development of various tumors and play a role in regulating the expression of target genes by pairing the 3′- untranslatable region with mRNA. Thus, miRNAs act as oncogenes or tumor suppressors [12].

Considering the important role of GPC3 and related miRNA regulation, it is important to identify GPC3-specific miRNAs. In Flora’s research, the expression of 10 miRNAs was related to GPC3 in HCC. Among them, miR-4510 was the top candidate miRNA, which was weakly expressed in HCC tissue, and its expression of miR-4510 was correlated with HCC cell proliferation and invasion [13]. In gastrointestinal stromal tumors, miR-4510 is expressed at low levels in tumor tissue, and the function of miR-4510 is related to the phosphorylation of protein kinase B and ERK1/2 (Extracellular-signal-Regulated kinase 1/2) [9]. However, in gastric adenocarcinoma, the expression of miR-4510 remains unclear. In this study, we first confirmed that miR-4510 was decreased in gastric cancer tissue compared with the adjacent normal tissue. Downregulation of miR-4510 could support gastric cancer cell proliferation, migration, and even in vivo metastasis. Thus, combining these results together, miR-4510 could function as a tumor suppressor in gastric cancer.

Besides miR-4510, the expression of GPC3 in gastric cancer is also controversial. In some studies, GPC3 was absent in invasive tumors and metastatic lymph nodes, and the presence of GPC3 causes downregulation of MAPK/FOXM1 (mitogen-activated protein kinase/forkhead box M1) signaling, which reduces the survival rate of gastric cancer patients [14]. In another study, overexpression of GPC3 expression could activate Wnt/β-catenin and further promote the proliferation and invasion of gastric cancer cells and reduce the apoptosis of gastric cancer cell lines [15].

In addition, it was reported that GPC3-GC was partially similar to hepatocellular carcinoma histologically, and functionally expressed albumin mRNA. Immunohistochemistry showed serum hepatoid marker alpha-fetoprotein (AFP) and GPC3 positivity in gastric cancer tissues from patients with elevated AFP and GPC3 [16]. Therefore, we speculated that some miRNAs in GPC3-expressing gastric cancer may also affect the proliferation of gastric cancer by regulating GPC3. We investigated the relationship between miR-4510 and GPC3 in GC using the luciferase reporter gene technique. The experiment found that GPC3 was downregulated by miR-4510 overexpression and that GPC3 acted as a target for miR-4510.

After identification of miR-4510 as a tumor suppressor in gastric cancer, we combined CyTOF, FACS, and IHC, and confirmed that the inhibition of miR-4510 increased the proportion of M2 macrophages in the tumor. In tumor development, the function of M2 macrophages is usually related to tumor progression and invasion, which are also classified as tumor-associated macrophages (TAMs) [17]. Activated TAM, which is characterized by the upregulation of ARG1 (Arginase-1), CD206, FIZZ1 (found in inflammatory zone 1), YM1 and so on [18], plays an important role in generating immunosuppressive TME by producing cytokines such as IL-4 and IL-6 [19], and growth factors such as insulin-like growth factor 1 (IGF1) and epidermal growth factor (EGF) [20]. We then tried to block the metastasis by drug treatment. We identified oxaliplatin as the candidate drug through the screening of drugs. Oxaliplatin is a well-known chemotherapeutic drug in gastric cancer, which been applied in the treatment of inoperable patients [21]. However, the resistance of oxaliplatin is unpredictable after treatment which might been caused by the change of AIF, P53, BRCA1 and so on [22]. In our study, we proved that the treatment of oxaliplatin could reverse the expression miR-4510 and modify the tumor microenvironment, which could guide the drug selection in gastric cancer.

In conclusion, our study demonstrated that GPC3 is a target gene of miR-4510, and downregulation of GPC3 could block the metastasis of gastric cancer cells caused by miR-4510 in vivo. Moreover the activation marker of M2 macrophages was highly increased, while the proliferation of CD8+ T cells was limited in the tissue and cell lines where miR-4510 had low expression, suggesting that the expression of miR-4510 can change the immunosuppressive signal in the tumor microenvironment. From the perspective of treatment, expression of miR-4510 and GPC3 was reversed by oxaliplatin treatment, which significantly reduced the proportion of M2 macrophages and restored the proliferation of CD8+ T cells, suggesting that oxaliplatin may become a specific therapeutic drug in patients with miR-4510 inhibition and GPC3-GC.

## Data Availability

Data will be made available on reasonable request.

## Abbreviations

HCC: Hepatocellular carcinoma
GC: Gastric cancer
GPC3: Glypican-3
GPC3‐GC: Glypican3-expressing gastric cancer
QPCR: Quantitative reverse transcription PCR
CyTOF: Cytometry by time-of-flight
FACS: Fluorescence activated cell sorting
IHC: Immunohistochemistry
MFC: Murine foregastric cancer cells
TAMs: Tumor-associated macrophages
TME: Tumor microenvironment
OE: Overexpressing
AGS: Human gastric adenocarcinoma
AFP: Alpha-fetoprotein
